# Proteomic analysis of tumor cell nuclear expulsion reveals significant cell adhesion and RNA binding programs in extracellular chromatin

**DOI:** 10.1038/s41598-025-11807-z

**Published:** 2025-08-01

**Authors:** Justin M. Gray, Woo Yong Park, Ronald J. Holewinski, Thorkell Andresson, Carmelo Carmona-Rivera, Mariana J. Kaplan, Li Yang

**Affiliations:** 1 Laboratory of Cancer Biology and Genetics, Center for Cancer Research, National Cancer Institute, National Institutes of Health, Bethesda, MD 20892 USA; 2 Department of Biology, Johns Hopkins University, Baltimore, MD 21218 USA; 3 Protein Mass Spectrometry Group, Center for Cancer Research, National Cancer Institute, National Institutes of Health, Frederick, MD 21701 USA; 4 Systemic Autoimmunity Branch, National Institute of Arthritis and Musculoskeletal and Skin Diseases, National Institutes of Health, Bethesda, MD 20892 USA; 5 Laboratory of Cancer Biology and Genetics, Center for Cancer Research, National Cancer Institute, National Institutes of Health, Building 37, Room 3134C, 37 Convent Drive, Bethesda, MD 20892 USA

**Keywords:** Cancer, Apoptosis, Chromatin, Proteomics, Nuclear expulsion, Breast cancer, Lung cancer, Cell death, Proteome informatics

## Abstract

Understanding mechanisms of cancer cell death and the resulting effect on disease progression is crucial in cancer biology and the insight will likely offer better options for therapeutic treatment. Nuclear expulsion occurs in apoptotic cancer cells in a peptidylarginine deiminase 4 (Padi4) dependent manner. The resulting tumor cell nuclear expulsion product (TuNEP) promotes the outgrowth of neighboring cancer cells through chromatin-bound protein complexes. It is not clear what the protein compositions and functionalities are in these TuNEPs. In this study, we performed extensive proteomic profiling and identified key TuNEP protein components from mouse and human breast cancer cells as well as human lung cancer cells (4T1, MDA-MB-231, and PC9). We further compared TuNEP- specific proteins with those from apoptotic bodies or NETs from neutrophils. We found an enrichment of cellular adhesion molecules as well as increased citrullination of proteins associated with RNA binding. We showed that cellular adhesion molecules integrin and basigin (BSG) promote the growth of tumor spheroids. Our work revealed the unique TuNEP protein components distinct from neutrophil-derived NETs and shed light on potential mechanisms by which these cancer cell-derived TuNEPs promote tumor progression.

**Supplementary Information:**

The online version contains supplementary material available at 10.1038/s41598-025-11807-z.

## Introduction

Apoptosis has been reported to be utilized by cancer for tumorigenic purposes^1–4^. We recently discovered that apoptosis induces peptidylarginine deiminase 4 (Padi4) dependent citrullination of histones and results in nuclear expulsion in tumor cells, which leads to the production of nuclear expulsion products (TuNEPs). TuNEPs activate RAGE, the receptor for advanced glycation end products through chromatin-bound RAGE ligands and enhance metastatic outgrowth of surrounding tumor cells^5^. Citrullination of histones leading to nuclear expulsion has been reported previously in neutrophils, a phenotype known as neutrophil extracellular traps (NETs)^6,7^. NETs are known to have proteases attached such as myeloperoxidase and neutrophil elastase, as well as other proteins like S100a8 and S100a9^8,9^. It is unclear whether tumor cell nuclear expulsion has similar protein components and pathways across different cancer types, as well as how TuNEPs and NETs differ in their protein contents.

Integrins are a group of adhesion molecules that form dimers and facilitate the interactions between the cells and their environment, such as the extracellular matrix (ECM)^10,11^. These interactions alter intracellular signaling and influence processes like cell division, survival, death, and disease progression, including cancer^12,13^. Notably, studies show that during apoptosis, integrins could be produced through extracellular vesicles (EVs, or apoEVs) particularly in association with apoptosis^14^. These secreted integrins play critical roles in tumorigenesis and metastasis by interacting with the microenvironment^11^.

Citrullination, also known as deimination, is a type of post-translational modification mediated by the peptidyl arginine deiminase (Padi) family. Citrullination exhibits distinct functions depending on the substrate being citrullinated and its specific role, such as in cell death or epigenetic regulation^15^. For example, neutrophils and tumor cells undergo nuclear expulsion via Padi4-mediated citrullination of histones, leading to processes such as neutrophil extracellular traps (NETs) during infection and tumor nuclear expulsion during metastasis or therapy^5–7^. Additionally, citrullination plays a significant role in gene regulation during developmental stages by modulating pluripotency through histone H1 citrullination^16^. Depending on the context, it can drive nuclear membrane disruption, regulate gene expression as an epigenetic modulator, or mediate the nuclear translocation of target substrates^17^. Recently, many citrullinated peptides beyond histones have been identified, and these modified proteins have been implicated in rheumatoid arthritis (RA) and are considered potential therapeutic targets^5,18–22^. In this study we conducted comprehensive analysis of proteomics of TuNEPs from 4T1 mouse mammary tumor cells, MDA-MB-231-LM3 human breast cancer cells, PC9 human lung cancer cells, as well as human NETs. We identified proteins as well as citrullinated proteins that are unique to TuNEPs.

## Materials and methods

### Cell lines

Murine 4T1 and human MDA-MB-231 breast cancer cell lines were purchased from the American Type Culture Collection (ATCC). A highly lung metastatic MDA231-lung met-3 (LM3) was also established from metastatic lung nodules by additional enrichment from MDA-MB-231LM2 and BrM2 cell line, which was gifted by J. Massague which was described previously^5^. PC9, a human lung adenocarcinoma cell line, was gifted by J. Amann and D. Carbone. Breast cancer cell lines and lung cancer cell lines were cultured in DMEM and RPMI-1640 respectively. Medium were supplemented with 10% heat-inactivated FBS, 500 U ml − 1 penicillin and 500 mg ml − 1 streptomycin at 37 °C in a humidified atmosphere containing 5% CO2. All cell was confirmed to be Mycoplasma negative.

### Generation of human NETs

Healthy controls were recruited through advertisements. Peripheral blood was obtained via venipuncture and collected into EDTA-containing tubes. The blood was then fractionated using a Ficoll-Paque Plus (GE Healthcare) gradient. Neutrophils were isolated by dextran sedimentation and hypotonic salt solution, following the previously described ( Carmona-Rivera et al. (Sci Immunol. 2017;2(10)). Ten million neutrophils from healthy volunteers were resuspended in RPMI and plated in 24-well plates with 2.5 µM of calcium ionophore (Sigma) for 4 h at 37 °C. Neutrophil extracellular traps (NETs) were harvested by treating with 10 U/mL of micrococcal nuclease (Thermo, Waltham, MD) for 15 min at 37 °C. The NETs were collected and cleared of debris by centrifugation at 5,000 rpm for 5 min at 4 °C. The supernatants were transferred to fresh Eppendorf tubes and stored at -20 °C.

### TuNEP and NET proteomics

Human tumor cell line TuNEP samples were prepared by serum starving the cells for 16 h and then plating 1 × 10^7^ in a 12 well plate. Neutrophils were isolated from human patients and plated at 1 × 10^7^ in a 12 well plate. Cells were then induced, with TuNEP samples being induced with 10 μm raptinal for 12 h and NETs induced using 5 μm ionophore for 4 h. 900uL of supernatant was removed and 300uL RPMI with 10 unites of MNase was added for 10 min. EGTA was added with a final concentration of 40mM to stop the reaction. The sample was centrifuged at 5,000 rpm for 5 min and the cleared supernatant was stored at − 80 °C.

Digestion and analysis of mouse 4T1 samples was performed as previously described^5^. For TuNEP samples pellets were lysed with EasyPep lysis buffer (Thermo #A45735) and protein concentration determined by BCA assay. For each sample 10 µg of each sample was treated with 50µL reducing solution and alkylation solution provided with Easypep Kit (Thermo #A40006) and incubated at 95 °C for 10 min and allowed to cool to room temperature before adding 2 µg of trypsin/LysC provided with the kit and incubated at 37 °C for 16 h. Samples were treated with 60 µg of TMTpro (Thermo #A52045) and incubated at room temperature for 1 h then quenched with 5% hydroxylamine, 20% formic acid an incubated for 10 min then combined. Combined TMTpro samples were cleaned using EasyPep mini spin columns provided with the kit. Eluted peptides were dried and then resuspended in 0.1% formic acid for LC/MS analysis using a Dionex U3000 RSLC in front of an Orbitrap Eclipse equpied with FAIMS interface and EasySpray ion source. Solvent A consisted of 0.1%FA in water and Solvent B consisted of 0.1%FA in 80%ACN. Loading pump consisted of Solvent A and was operated at 7 µL/min for the first 6 min of the run then dropped to 2 µL/min when the valve was switched to bring the trap column (Acclaim™ PepMap™ 100 C18 HPLC Column, 3 μm, 75 μm I.D., 2 cm, PN 164535) in-line with the analytical column EasySpray C18 HPLC Column, 2 μm, 75 μm I.D., 25 cm, PN ES902). The gradient pump was operated at a flow rate of 300nL/min and each run used a linear LC gradient of 5–7%B for 1 min, 7–30%B for 133 min, 30–50%B for 35 min, 50–95%B for 4 min, holding at 95%B for 7 min, then re-equilibration of analytical column at 5%B for 17 min. All MS injections employed the TopSpeed method with three FAIMS compensation voltages (CVs) and a 1 s cycle time for each CV (3 s cycle time total) that consisted of the following: Spray voltage was 2200 V and ion transfer temperature of 300 ⁰C. MS1 scans were acquired in the Orbitrap with resolution of 120,000, AGC of 4e5 ions, and max injection time of 50ms, mass range of 350–1600 m/z; MS2 scans were acquired in the Orbitrap using TurboTMT method with resolution of 15,000, AGC of 1.25e5, max injection time of 22ms, HCD energy of 38%, isolation width of 0.4Da, intensity threshold of 2.5e4 and charges 2–6 for MS2 selection. Advanced Peak Determination, Monoisotopic Precursor selection (MIPS), and EASY-IC for internal calibration were enabled and dynamic exclusion was set to a count of 1 for 15 s. The only difference in the methods was the CVs used, one method used CVs of -45, -60, -75, method 2 used CVs − 50, -65, -80, and method three used CVs of − 55, − 70, − 85.

All injections from each experiment were batched together as fractions and all MS files were searched with Proteome Discoverer 2.4 using the Sequest node. For all experiments the data was searched against the Uniprot Mouse database or Human database from August 2020 using a full tryptic digest, 2 max missed cleavages, minimum peptide length of 6 amino acids and maximum peptide length of 40 amino acids, an MS1 mass tolerance of 10 ppm, MS2 mass tolerance of 0.02 Da, variable oxidation on methionine (+ 15.995 Da), variable deamidation on Arg, Gln, Asn (+ 0.9884 Da), fixed TMTpro (+ 304.207) on lysine and peptide N-terminus, and fixed modification of carbamidomethyl on cysteine (+ 57.021). Percolator was used for FDR analysis and only proteins FDR cutoff was set to 1%. TMTpro reporter ions were quantified using the Reporter Ion Quantifier node and normalized on medain intensity of each channel and no imputation was used. All RAW mass spectrometry files are available on the MassIVE database with accession number MSV000097089.

### Proteomic analysis

For all experiments altered protein expression and protein citrullination in various comparisons used Log2FC > 0.6 and p value < 0.05), Xcorr > 1.7, and with unambiguous localization. Citrullinated proteins were considered as high confidence with site localization scores > 90, no N or Q adjacent flanking R. For medium confidence: site localization scores > 90, N or Q adjacent flanking R.

For low confidence: citrullinated R on C terminus or multiple Citrullinations assigned to same R or no score on R. Protein network analysis performed using STRING. Proteins associated with the RNA binding was analyzed using GO molecular function and are highlighted in red when FDR = 8.30e−06.

Heatmaps were generated with Morpheus (software.broadinstitute.org/morpheus/), and the top variable genes, up or down regulated were displayed. GSEA (v4.2.1) was used for pathway analysis and the top 1,000 most variable proteins from Morpheus were used for this analysis. The top upregulated genes from GSEA’s ranked list were then used to create STRING (https://string-db.org/) protein interaction networks. Venn diagrams were made using the gplots package in Rstudio.

### Immunofluorescence of TuNEPs

To generate TuNEPs, the cells were starved for 16 h beforehand, followed by 2 × 10e6 cells in a 12 well with raptinal treatment for 12 h to induce nuclear expulsion. The sample was centrifuged at 500 g to get rid of intact cells. The supernatant was then centrifuged at 16,000 g and resuspended in 100uL 1% BSA in PBS.

For immunofluorescence of TuNEPs, conjugated primary antibodies were added for 1 h. TuNEPs were then fixed in 2% PFA for 10 min and centrifuged at 16,000 g for 10 min followed by a PBS wash and centrifugation again. Intact nuclear expulsion samples were then added to a 96 well glass plate and centrifuged at 1,000 g for 10 min and imaged. GFP labelled H2B for PC9 and 4T1 cells as well as mChemrry labelled H2B for MDA-MB-231 cells were used to stain nuclear contents. To match the color of nuclear contents among different cell lines, pseudo color of green for mCheery was used.

To examine EVs, anti-CD9 stained but not fixed TuNEPs were treated with 30U of MNase for 20 min at 37 °C. The sample was then centrifuged at 2,000 g to get rid of debris. The supernatant was collected and centrifuged at 20,000 g to pellet large EVs. The remaining supernatant was then centrifuged at 100,000 g at 4 °C for 70 min to pellet small EVs. EVs were resuspended in 100uL and 10uL was added to each well of a glass 96 well and centrifuged at 1,000 g for 10 min. The samples were then imaged. Confocal images were taken on a Nikon SoRa Spinning Disk with a Photometrics BSI sCMOS camera at 20x and 60x resolution for intact TuNEPs and EVs respectively.

### Spheroid culture

2,000 tumor cells were added to a U-bottom ULA plate in 2% FBS in Fluorobrite DMEM and the plate was centrifuged at 400 g for 10 min. The cells were grown for 3 days in the presence of drugs. The media was removed and 40uL of 75% Matrigel was added, and the spheroids were centrifuged at 400 g for 10 min. Matrigel was solidified at 37^o^C for 35 min. 100uL of 2% FBS Fluorobrite media with indicated drugs was added to the top. Media was changed every three days. After 12 days the size of the spheroids was calculated using ImageJ.

### Preparation of NEPs or apoptotic debris (ApoDBs) from tumor cells

Cells were cultured in DMEM + 10% FBS and allowed to grow to 90% confluence followed by serum starvation for 16 h. Cells were detached, washed with PBS three times and were then resuspended with 1 ml 1 mM CaCl_2_ containing RPMI and plated at a concentration of 1 × 10^7^ per ml on a 12-well plate. To prepare non-toxic dead contents, inducible caspase-9 (iCasp9) system, consisting of an FKBP12-F36V dimerizing domain fused with caspase-9 and induced by AP1903, was used^5^. With a treatment of 8 nM AP1903, iCasp9-transduced MDA-MB-231 LM3 or Par cells were used for enriching NEPs or apoDBs, respectively. NEPs or apoDBs were added to recipient cells in the following amounts, co-culture (NEPs/apoDBs from 5,000 cells to 500 recipient cells).

### Cell proliferation, viability and adhesion when co-cultured with NEPs

Tumor cells (5 × 10²) were seeded in triplicate into 96-well plates, and cell viability was assessed after 2 days using the CellTiter-Glo Luminescent Cell Viability Assay (Promega, G7572), following the manufacturer’s instructions. For co-culture experiments, 5 × 10² MDA-MB-231 parental cells were plated and allowed to adhere for 24 h. NEPs or ApoDBs, generated via the iCasp9 system, were then added in DMEM supplemented with 0.2% FBS. To investigate downstream signaling pathways, either TC-15-I (10 µM) or SP-8356 (10 µM) was added to the culture medium 2 h prior to NEP treatment.

## Results

### Proteomic analysis of human tumor cell nuclear expulsion products (TuNEPs)

**Fig. 1:**
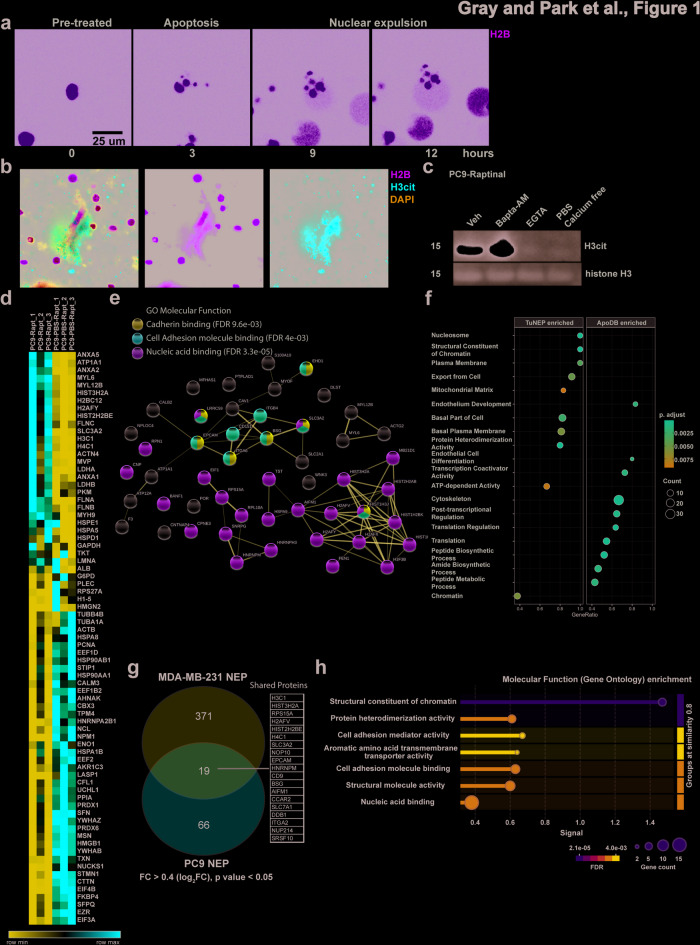
Proteomic analysis of tumor cell nuclear expulsion products (TuNEPs) from PC9 human lung adenocarcinoma (LuAC). (**a**) live cell imaging of nuclear expulsion of Raptinal treated PC9. (**b**) immunofluorescence (IF) of TuNEPs of PC9. Red: CitH3, Green: H2B: GFP, Blue: DAPI. (**c**) western blots of citrullinated histone H3 (CitH3) and total histone H3 (histone H3) from PC9 cells in culture treated with 10 μm Raptinal. (**d–f**) heatmap of the top 75 most differential proteins (**d**), GO pathway (**e**) and STRING network (**f**) analysis from PC9 derived TuNEPs compared with ApoDBs. (**g**) Venn diagram analysis for TuNEP proteins comparing MDA-MB-231-LM3 vs. PC9. (**h**) GO Pathway analysis of shared TuNEP components obtained from both MDA-MB-231-LM3 and PC9. We performed unbiased pathway analysis using STRING with the following criteria: maximum FDR less than 0.05, minimum interaction strength greater than 0.01, minimum signal intensity above 0.01, and a minimum of two proteins per network. The top-listed Gene Ontology (GO) terms from this analysis are presented. Significant FDR values are shown.

Our previous studies highlighted that nuclear expulsion occurs in a variety of cancer cells^5^. Here, we sought to compare TuNEPs across different cancer types to understand the common and differential TuNEP protein components. PC9, derived from human lung adenocarcinoma (LuAC), underwent nuclear expulsion and produced web-like citrullinated chromatin when treated with apoptosis-inducing reagent raptinal (Fig. 1a and b). As expected, cultured PC9 cells with treatment of apoptosis inducer Raptinal showed an increase in citrullinated histone H3 (H3cit), a marker of tumor nuclear expulsion (TuNE). PC9 treated with EGTA, a calcium chelator, and PC9 culture with calcium-free PBS, which lacks of *extracellular* calcium, effectively abrogated H3 citrullination, suggesting Ca^++^ dependent TuNE and is consistent with our previous publication (Fig. 1c). Interestingly, addition of BAPTA-AM, a cell-permeable calcium chelator, did not suppress H3cit, perhapse due to inefficient *intracellular* calcium chelation under the particular culture context of PC9 cells (Fig. 1c).

We performed proteomic analysis comparing TuNEPs and apoptotic bodies that were collected from PC9 cell culture in PBS (without calcium to induce apoptosis and without nuclear expulsion). Of the 1,491 proteins identified, 1,241 have 2 or more PSMs (Peptide-Spectrum Matchs) (Fig. S1a and b). As expected, histone proteins were enriched in TuNEPs including HIST3H2A, H2BC12, H2AFY, HIST2H2BE, H3C1, and H4C1 (Fig. 1d). In addition, ANXA proteins were abundant in PC9 TuNEPs including ANXA1, ANXA2, and ANXA5. STRING, GO and KEGG pathway analysis revealed that TuNEP-abundant proteins were primarily associated with chromatin pathways, but excluding chromatin proteins, cell adhesion molecules emerged as the most prominent category, clearly distinguishing them from apoptotic debris (ApoDBs, Fig. 1e–f, and Fig. S1c). The cell adhesion molecule binding cluster in TuNEPs contained tumorigenic proteins such as ITGA2, CD151, CD9, SLC3A2, and BSG, all known to have an impact on the tumor microenvironment (Fig. 1d and e). Interestingly, CD9 and CD151 are also a marker for EVs, indicating that TuNEPs may contain EV protein components.

A different protein profile of TuNEPs from lung cancer cells was observed when compared with those from breast cancer cells. Of particular note, TuNEPs derived from PC9 cells lacked HMG family proteins and S100a4, which were identified in TuNEPs from human MDA-MB-231 LM3 and mouse 4T1 breast cancer cells, and were critical for their roles in metastatic outgrowth^5^. As expected, core chromatin-related proteins, including histones, and cell adhesion proteins were specifically found in TuNEPs from both cancer types (Fig. 1g–h and Figure S1d–e). Together, these data show a differential TuNEP protein profile between human lung and breast cancers, yet a similar TuNEP protein profile between human and mouse breast tumors as previously shown, with a consistent enrichment of cell adhesion proteins among them.

### Protein citrullination of lung and breast cancer TuNEPs

**Fig. 2:**
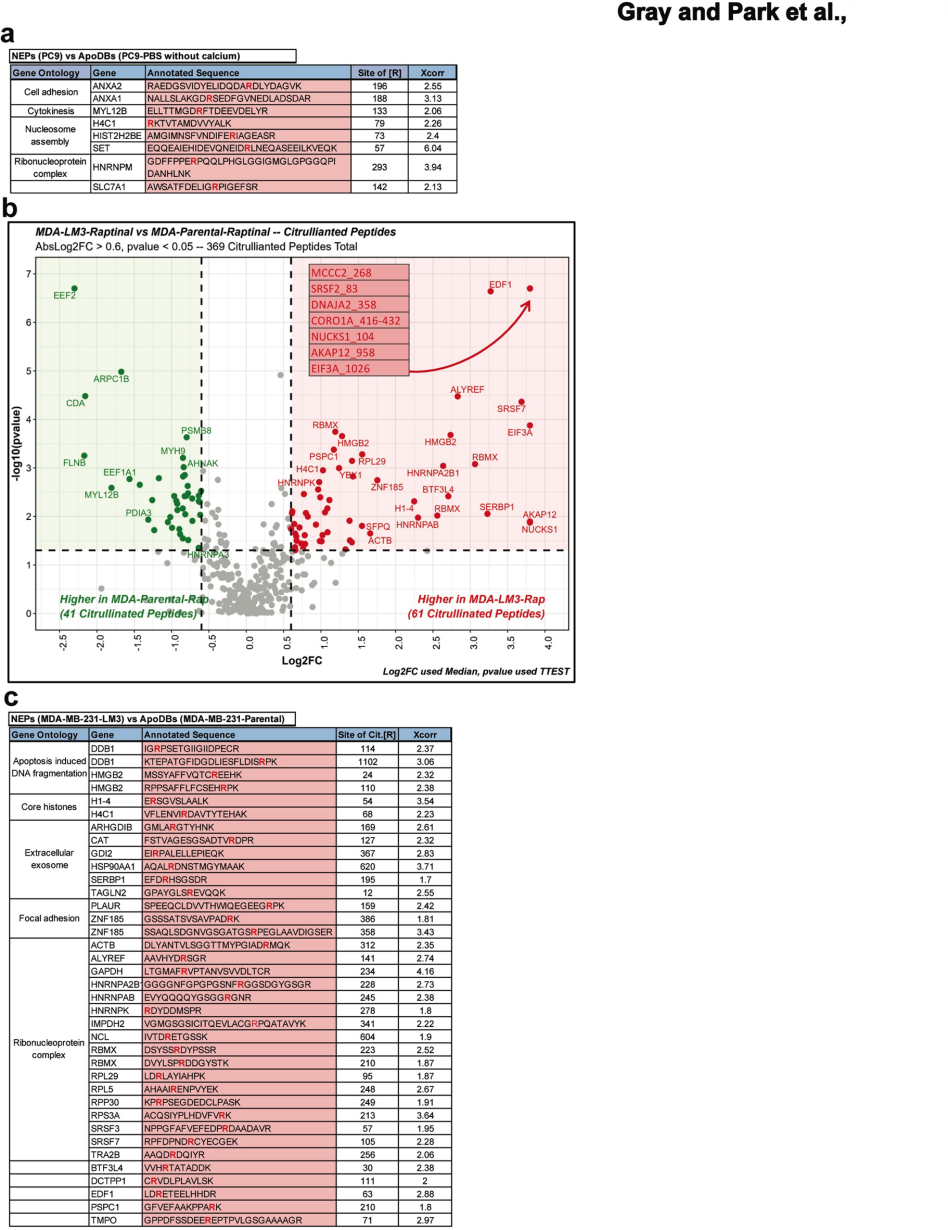
Profiling of citrullinated proteins in lung and breast cancer TuNEPs. (**a**) list of citrullinated peptides in TuNEPs compared with ApoDBs from PC9 cells with calcium- vs. without calcium-PBS respectively. (**b** and **c**) volcano plot (**b**) and list (**c**) of differential citrullinated peptides in TuNEPs compared with ApoDBs from MDA-MB-231-LM3 vs. MDA-MB-231 parental cells respectively. 10 μm Raptinal treated. For volcano plot, AbsLogFC > 0.5, *p* < 0.05, 369 citrullinated peptides with 1 peptide, 1%FDR. For list, changes are Log2FC > 0.6 and p value < 0.05), Xcorr > 1.7, and with unambiguous localization. Citrullinated proteins in high confidence were called when site localization scores > 90, no N or Q adjacent flanking R, or with medium confidence with site localization scores > 90, N or Q adjacent flanking R.

Histone citrullination is a hallmark of nuclear expulsion in both tumor cells and neutrophils. In addition, citrullination of various protein has been implicated in auto-immune disease and inflammatory conditions^5,18–21^. To investigate protein citrullination in TuNEPs, we first profiled citrullinated peptides from TuNEPs derived from PC9 and MDA-MB-231 LM3 cells. Using stringent criteria (log₂ fold change > 0.6, p-value < 0.05, Xcorr > 1.7, and unambiguous site localization), we identified a total of 229 citrullinated peptides in PC9-derived TuNEPs and 424 citrullinated peptides in TuNEPs from MDA-MB-231 LM3 cells. In PC9 derived TuNEPs, core histones, such as H4C1, and ANXA proteins were found to be citrullinated (Fig. 2a and Figure S2a). However, the overall number of proteins detected as citrullinated was significantly lower compared to TuNEPs derived from MDA-MB-231 LM3 cells. In TuNEPs derived from MDA-MB-231 LM3, when compared with ApoDBs from MDA-MB-231 parental, not only histones such as H1-4 and H4C1, but also apoptosis- and ribosomal complex- related proteins were found to be citrullinated (Fig. 2b, c and Figure S2b). Interestingly, 54 residue of histone H1, which is known to induce pluripotency and prevents binding of H1 to the nucleosome^16^was strongly citrullinated during nuclear expulsion in MDA-MB-231 LM3 model. Consistently, the mouse 4T1 TuNEPs showed particularly enriched citrullination of histone proteins including H2afx, H4C1, Hist1h2bb, Hist2h2bb, H3c1, Hist2h2ac, H1-2, H1-4, and H1-5 when compared to the ApoDBs (Figure S2c). However, there appeared to be very few shared citrullinated proteins between the breast and lung cancer TuNEPs (Fig. 2a-c and Figure S2d), suggesting the existence of a cancer-specific protein citrullination profile during nuclear expulsion.

Of great interest, there were four proteins, ALYREF, HNRNPAB, LMNA and GAPDH, shared by all three comparisons (Fig. S2d). ALYREF and HNRNPAB are RNA-binding and RNA-processing-related proteins, while LMNA and GAPDH are housekeeping proteins involved in cell shaping. However, when present in the extracellular space, these proteins are well-known DAMPs (damage-associated molecular patterns), suggesting that TuNEPs may serve as a primary source of DAMPs regardless of their origin or species^5^.

### TuNEP protein profiling and citrullination compared with NETs

Tumor nuclear expulsion and NET formation share many similarities, particularly in their mechanism relying on citrullination and chromatin expulsion. However, considering their context-specific roles and distinct cellular origins, the composition of the complexes is likely to differ significantly. Our previous studies have shown that, apart from the chromatin forming their structural framework, the specific compositions of TuNEPs include DAMPs such as S100a4 and HMGs, which is different from those of NETs which are characterized by proteases like myeloperoxidase and neutrophil elastase^5,23,24^. Here we performed a detailed comparison of protein profiling and peptide citrullination. We compared TuNEPs derived from human breast cancer MDA-MB-231-LM3 cells with human NETs, side by side, under A23187 calcium ionophore treatment in both tumor cells and neutrophils. In the MDA-MB-231 LM3 dataset, a total of 2,525 proteins were identified, among which 1,990 proteins had two or more peptide-spectrum matches (PSMs).

**Fig. 3:**
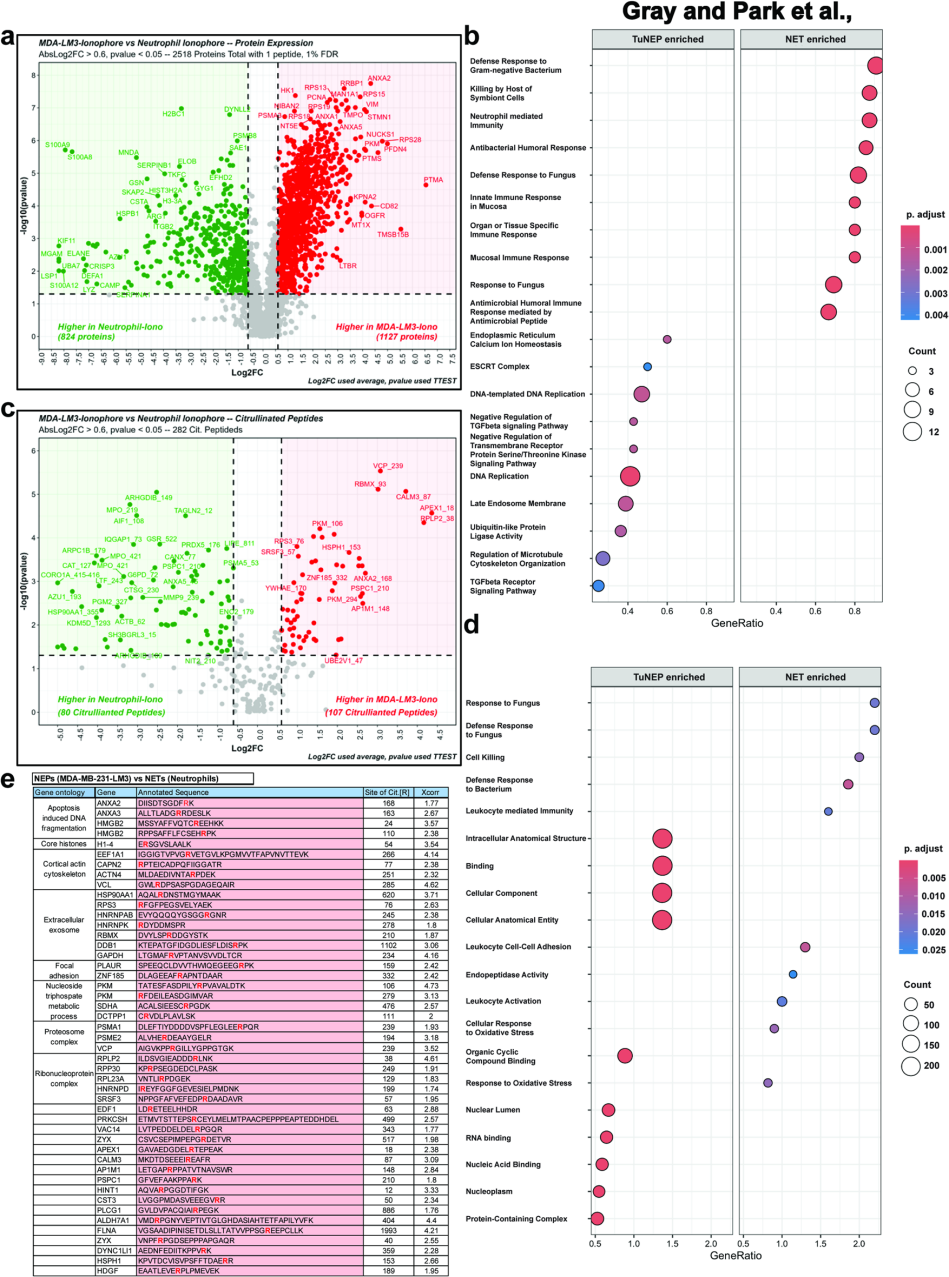
Profiling of TuNEP-abundant proteins and citrullination compared to NETs. (**a** and **b**) volcano plot (**a**) and GO pathway analysis (**b**) of differential proteins from MDA-MB-231 LM3 derived TuNEPs compared with neutrophil derived NETs. (**c**–**e**) volcano plot (**c**), GO pathway analysis (**d**), and list (**e**) of differential citrullinated peptides from MDA-MB-231 LM3 derived TuNEPs compared with neutrophil derived NETs. 5 μm A23187 ionophore treated. For volcano plot, AbsLogFC > 0.5, *p* < 0.05, 2430 of whole proteins and 361 of citrullinated peptides with 1 peptide, 1%FDR. For list, changes are Log2FC > 0.6 and p value < 0.05), Xcorr > 1.7, and with unambiguous localization. Citrullinated proteins in high confidence were called when site localization scores > 90, no N or Q adjacent flanking R, or with medium confidence with site localization scores > 90, N or Q adjacent flanking R.

For total protein profiling specific to TuNEPs, the top differential proteins such as VIM and ANXAs were identified in the volcano plot (Fig. 3a). GO pathway analysis using GSEA revealed that TuNEPs were enriched in pathways related to ESCRT (endosomal sorting complex required for transport; a key mediator of multivesicular body biogenesis), DNA-binding proteins, and TGF-beta signaling (Fig. 3b). In contrast, NETs exhibited enrichment in pathways associated with immune responses. KEGG pathway analysis also showed distinctive profile of TuNEP from NETs, exhibiting cytoskeletons and DNA repair pathways (Figure S3a). These findings highlight significant differences in the biological pathways between TuNEPs and NETs, reflecting their distinct functional roles. Furthermore, tumor-specific NEP proteins comparing LM3 TuNEPs with NETs were further validated through cross-referencingwith proteins from apoDB, generated from Raptinal-induced apoptosis or A23187-induced directed nuclear expulsion (Figure S3b). These results suggest a unique TuNEP protein profile that’s different from that of NETs or the byproducts of apoptosis.

For TuNEP specific peptide citrullination compared with NETs, we found various proteins such as HMGs, H1-4, RBMX, VCL and PLAUR which were associated with multiple pathways including apoptosis, histones, exosomes, cytoskeleton and focal adhesion (Fig. 3c-e). In contrast, NETs exhibited distinct citrullinated peptides, including MPO and MMP9 (Fig. 3c). Additional KEGG analysis by GSEA comparing TuNEPs versus NETs revealed citrullination of focal adhesion and cytoskeleton related proteins (Figure S3c). These data suggest that various proteins were citrullinated in tumor-derived TuNEPs, underscoring the need for further investigation of the distinctive functionality of TuNEP protein citrullination.

### Co-localization of cell adhesion proteins with TuNEPs

**Fig. 4:**
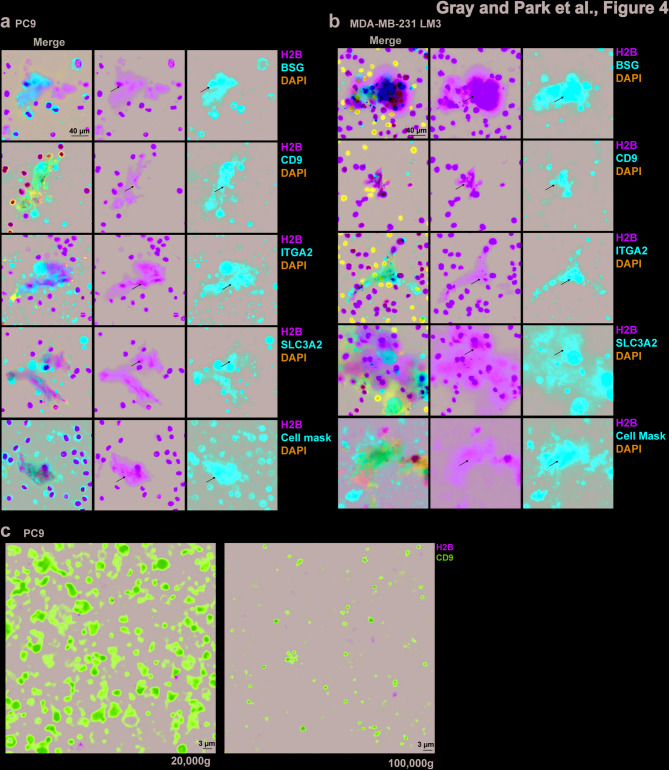
Immunofluorescence staining of cell adhesion proteins and TuNEPs. (**a**) IF of cell adhesion proteins (red) BSG, CD9, ITGA2, and SLC3A2 in TuNEPs from cultured PC9 cells. The H2B: GFP (green) marking chromatin. Cell mask red is a dye that stains the cellular membrane. (**b**) IF of cell adhesion proteins (pseudo Red) with H2B: mCherry (pseudo Green) marking chromatin in TuNEPs from cultured MDA-MB-231-LM3 cells. (**c**) IF of large (**Left**) and small (**Right**) EVs from PC9 TuNEPs isolated by differential centrifugation (20,000 g and 100,000 g respectively).

Our proteomic results indicate that a cluster of cell adhesion proteins are likely bound to TuNEPs. To further validate this finding, we stained these protein candidates in TuNEPs isolated from PC9 and MDA-MB-231 LM3 cells in culture treated with Raptinal. TuNEPs were clearly visible as dispersed H2B from PC9 and MDA-MB-231-LM3 cells (Fig. 4a-b, and Figure S4a), as well as from 4T1 cells (Figure S4b andc). BSG was found to colocalize with dispersed TuNEPs in the extracellular space and exhibited clear membrane staining in intact cells (Fig. 4a-b, arrow). ITGA2 and SLC3A2 also showed a strong overlap in extracellular space with TuNEPs, showing a greenish white signals due to strong GFP (Fig. 4a-b, arrow). Using cell mask red, a dye that stains the membrane, we also found that TuNEPs contain lipid membranes in MDA-MB-231-LM3 and PC9 TuNEPs (Fig. 4a-b, last row). Consistently, the MDA-MB-231-Parental and 4T1 Padi4^KO^ cells, which do not go through nuclear expulsion, did not show the overlap of the adhesion proteins with TuNEPs (Figure S4a-c). Interestingly but expected from the proteomics analysis, CD9, the common marker for EVs, was also found to overlap with TuNEPs in all samples (Fig. 4a-b, second row).

Considering the cell adhesion proteins are mostly membrane bound, and the fact that EV marker CD9 was found in TuNEPs, we sought to determine if EVs were attached to TuNEPs. We purified the TuNEPs, treated them with MNase, and centrifuged the remnants first at 500 g to remove cell debris and then at 20,000 g and 100,000 g to purify large EVs and small EVs respectively. Notably, we found CD9 + EVs in PC9 TuNEPs that lacked H2B: GFP signal with the 20,000 g sample containing larger EVs and the 100,000 g sample containing small EVs (Fig. 4c). These data suggest that TuNEPs likely trap the EVs. In summary, these findings indicate that cell adhesion molecules are enriched in TuNEPs which could be acting as a scaffold complex to facilitate tumor progression.

### BSG and ITGA2, cell adhesion molecules from TuNEPs, and enhanced tumor growth

**Fig. 5:**
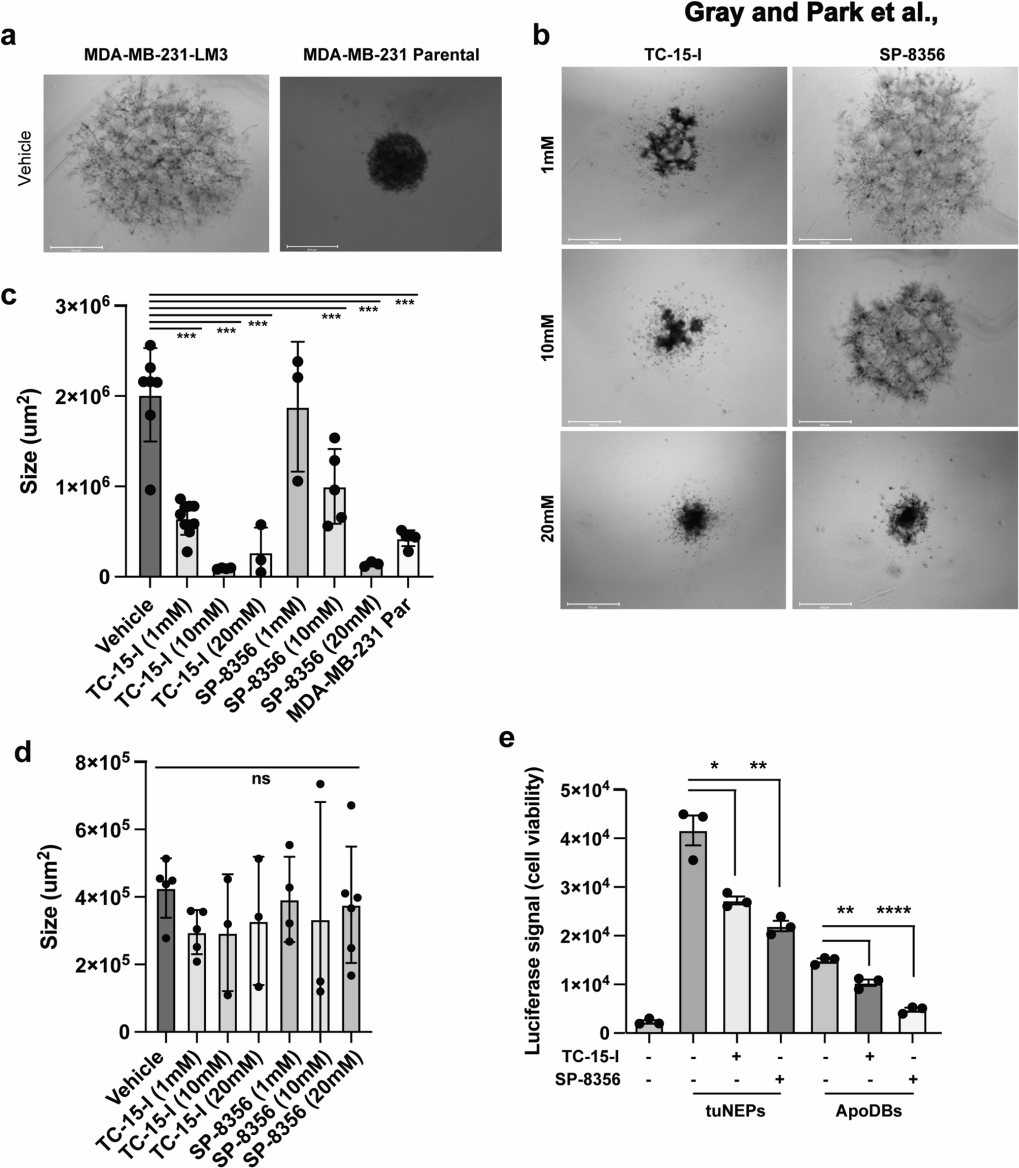
Basigin and Integrin A2 in TuNEP mediated tumor outgrowth. (**a**) Representative images of spheroids from MDA-MB-231-LM3 and parental MDA-MB-231-Par cells cultured for 12 days. (**b**) Representative images of MDA-MB-231-LM3 spheroids treated with ITGA2 and BSG inhibitors TC-15-I and SP-8356, respectively. (**c**) Quantification of the size of MDA-MB-231-LM3 derived spheroids treated with TC-15-I and SP-8356 compared to MDA-MB-231-Par spheroids. (**d**) Quantification of the size of MDA-MB-231-Par spheroids treated with TC-15-I and SP-8356. (**e**) Luciferase signals from the co-culture of MDA-MB-231-Par cells with TuNEPs or apoDBs that were treated with TC-15-I and SP-8356. CTG assay was used for measuring cell viability through luciferase signal.

Our proteomic and IF findings demonstrate that TuNEPs have enriched cell adhesion proteins BSG and ITGA2, we next investigated whether these proteins impact tumor growth. We cultured MDA-MB-231-LM3 spheroids and found that they invaded Matrigel and formed large spheroids, whereas the parental line MDA-MB-231 spheroids did not invade and remained small (Fig. 5a). When treated with TC-15-I and SP-8356, the ITGA2 and BSG inhibitors respectively, there were growth inhibition of MDA-MB-231-LM3 spheroids in a dose dependent manner, with no effect on the spheroid growth of parental MDA-MB-231-Par cells (Fig. 5b-d). This indicates that these cell adhesion proteins are vital to growth and invasion of the spheroids into the Matrigel. In addition, in a 2D co-culture assay of MDA-MB-231-Par tumor cells with isolated TuNEPs or apoDBs (apoptotic debris, as a control) in starvation condition with 0.2% FBS containing DMEM medium, there was a TuNEPs mediated growth increase which was ablated when treated with TC-15-I and SP-8356 (Fig. 5e). Compared with TuNEPs, ApoDBs triggered less growth in the MDA-MB-231-Par cells, and this was also minimized when treating with TC-15-I and SP-8356. Together these data suggest that TuNEPs induce enhanced outgrowth of tumor cells through adhesion proteins BSG and ITGA2.

## Discussion

Our protein profiling and citrullination analysis of TuNEPs has revealed pathways specific to tumor cell nuclear expulsion, showing similarity between mouse and human breast cancer cells, as well as evident difference between breast and lung cancer cells. Notably, a cluster of enriched cell adhesion proteins was upregulated in TuNEPs but not found in apoptotic bodies or NETs. Our studies further show that integrin and basigin in TuNEPs promote the spheroid growth of cancer cells. These findings enhanced our understanding of tumor cell nuclear expulsion and the potential mechanisms underlying extracellular chromatin’s role in cancer progression and metastasis relapse.

Understanding the compositions of TuNEPs from different cancers is vital to determining the overall impact of nuclear expulsion in cancer. Interestingly, the MDA-MB-231-LM3 breast cancer TuNEPs exhibited a group of RAGE agonists, including HMGB1 and HMGB2, which were absent in PC9 lung cancer TuNEPs. This suggests that RAGE signaling may be specific to breast cancer NEPs, consistent with our previous report on the 4T1 TuNEPs^5^. More importantly, several cell adhesion proteins, such as BSG, ITGA2, CD9, SLC3A2 or CD151, were significantly enriched in TuNEPs and shared across cancer types. BSG, which consists of SLC3A2 and SLC7A5, is known to colocalize and enhance tumorigenesis in hepatocellular carcinoma^25–27^. Additionally, BSG s associated with poorer patient outcomes in non-small cell lung cancer^25–27^. CD98, BSG, CD9 and CD151 have all been shown to interact with many of these interactions driving tumorigenesis^28–31^. Furthermore, MDA-MB-231-LM3 spheroids grew larger than MDA-MB-231-Par spheroids; however, blocking either Itga2 or Besigin significantly reduced MDA-MB-231-LM3 spheroid growth and had no effect on MDA-MB-231-Par spheroids. This indicates that the cell adhesion proteins are crucial for enhancing tumor growth and may interact with TuNEP to facilitate this process.

TuNEPs harbor adhesion markers, including CD9^32^, which are also found in EVs and microvesicles, but appear more abundant in TuNEP compared to NETs. Notably, apoptosis results in the formation of apoptotic extracellular vesicles (apoEVs) which include apoptotic bodies (ApoBDs)^14^microvesicles (ApoMVs)^33^and exosomes (ApoExos)^34^. Our previous findings indicate that apoptotic body formation precedes nuclear expulsion, implying that TuNEPs likely incorporate various vesicles. This raises the possibility that TuNEPs may serve as a key mechanism for delivering EVs into the extracellular space. Further studies are warranted to investigate whether this process influences the functional impact of EVs. Consistent with this, our analysis also revealed an upregulated exosome pathway in human breast and lung cancer TuNEPs when we examined shared citrullinated peptides, which was not found in NETs.

Another interesting group of molecules in TuNEPs are splicing factors. A recent study highlighted that ApoEVs can carry splicing factors that promote glioblastoma cells malignancy^35^. Similarly, we identified several splicing proteins, such as SNRPD2, SNRPG, SNRPD3, HNRNPM, HNRNPH3, and SRSF10, in human breast and lung cancer TuNEPs but not in NETs. Pathway analysis revealed spliceosomal snRNP assembly and U4 snRNP as common pathways in breast and lung cancer TuNEPs. Additionally, numerous splicing pathway proteins were also including ALYREF and HNRNPA2B1, were citrullinated. Further studies are needed to elucidate how citrullination affects their roles in tumorigenesis and metastasis.

Finally, the peptide citrullination unique to TuNEPs is particularly interesting. We observed more citrullinated peptides in the MDA-MB-231 LM3 model compared to the PC9 lung cancer model, potentially due to higher Padi4 levels in MDA-MB-231 LM3 cells. We suspect that elevated Padi4 activity might drive greater diversity in citrullination. Expanding the analysis to additional TuNEP models, including EO771-LM, bladder cancer cell lines like RT-112, and SW-760, could strengthen these findings. Nevertheless, the changes in citrullination composition not only highlight its potential as a biomarker for detecting TuNEPs but also underscore its broader role in regulating protein-protein interaction, stability, and activity.

Citrullination, as a post-translational protein modification, is known to regulate epigenetic processes and the nuclear translocation of substrates within cells. A notable example of citrullination’s role in epigenetic regulation is its mechanism of competing with histone methylation on arginine residues^36^. During cell death, particularly in processes like NET or TuNEP, padi4 citrullinates various proteins, including histones. However, the roles and functions of citrullination on non-histone peptides, especially extracellularly exposed proteins such as citrullinated VIM or citrullinated ITGA, remain largely unexplored^15,16^. Of note, elevated levels of citrullinated proteins, such as histones and vimentin, have been linked to rheumatoid arthritis (RA), suggesting that citrullination may contribute to the pathological implications of RA and cancer^37^. Future studies of TuNEP protein citrullination shall provide insight for nuclear expulsion and extracellular chromatin in cancer biology.

## Electronic supplementary material

Below is the link to the electronic supplementary material.

Supplementary Material 1

Supplementary Material 2

Supplementary Material 3

Supplementary Material 4

Supplementary Material 5

Supplementary Material 6

Supplementary Material 7

Supplementary Material 8

Supplementary Material 9

Supplementary Material 10

Supplementary Material 11

Supplementary Material 12

Supplementary Material 13

Supplementary Material 14

## Data Availability

The proteomics dataset has been deposited in MassIVE and will remain private until publication. Data from PC9, MDA-MB-231, and neutrophil experiments are accessible under the MassIVE accession number MSV000097089, while data from 4T1 have been deposited under MSV000087691, as previously described⁵. Login credentials for accessing the dataset for PC9, MDA-MB-231, and neutrophil experiments are provided below. Accession: MSV000097089Reviewer username: MSV000097089_reviewerPassword: YangAdditional information required for reanalysis of the reported data is available from the lead contact, Li Yang (yangl3@mail.nih.gov) upon request.

## Materials availability

All reagents generated in this study (including cell lines and plasmids) are available on request to L. Y.
