# Supplementary material for: Proteomic analysis of tumor cell nuclear expulsion reveals significant cell adhesion and RNA binding programs in extracellular chromatin
Source: Sci Rep. 2025 Aug 1;15:28054. doi: 10.1038/s41598-025-11807-z (PMC12317086; doi:10.1038/s41598-025-11807-z)

# Raw data of western blots

Figure. 1c

**samples were prepared using acid extraction (following the Abcam protocol), which leaves only histones**

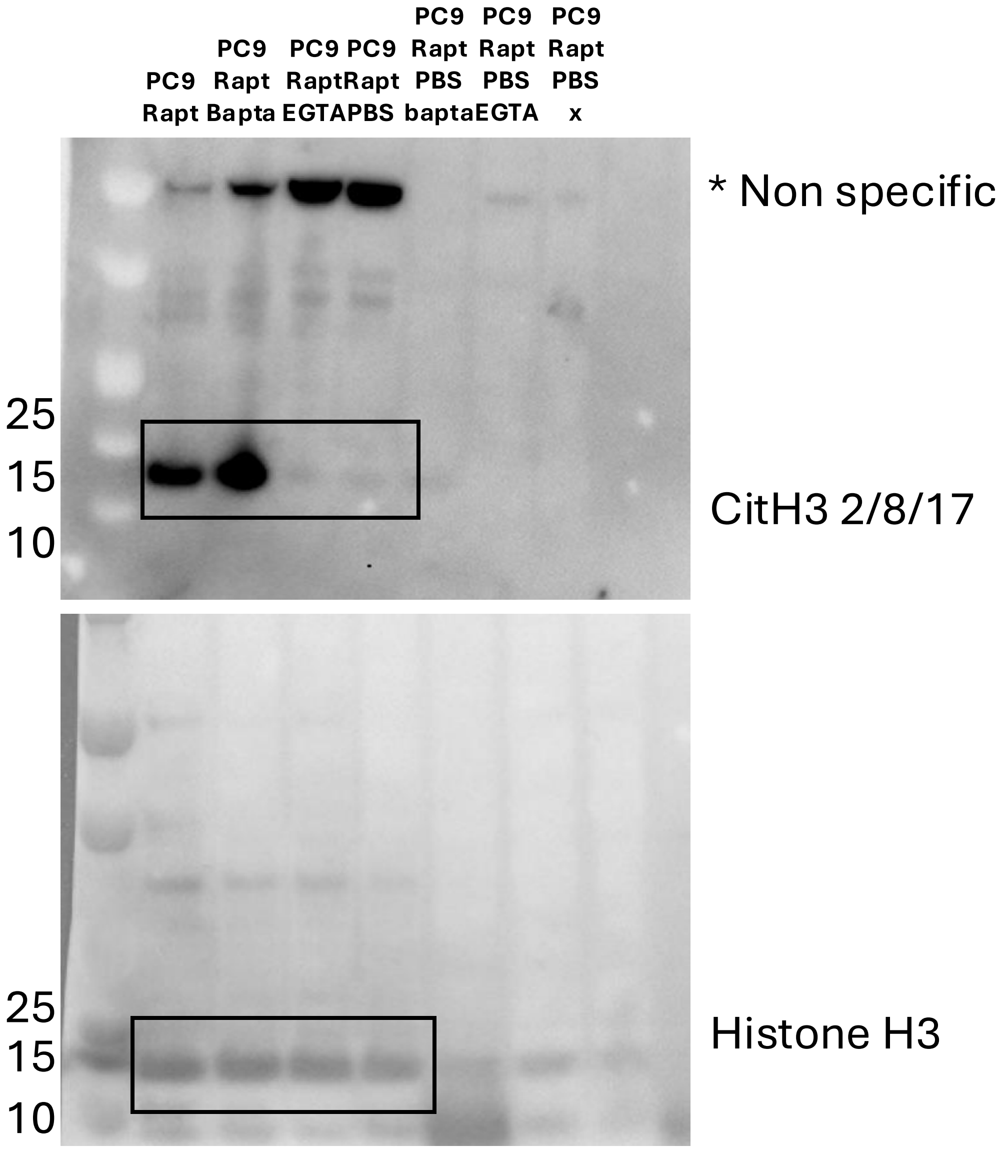


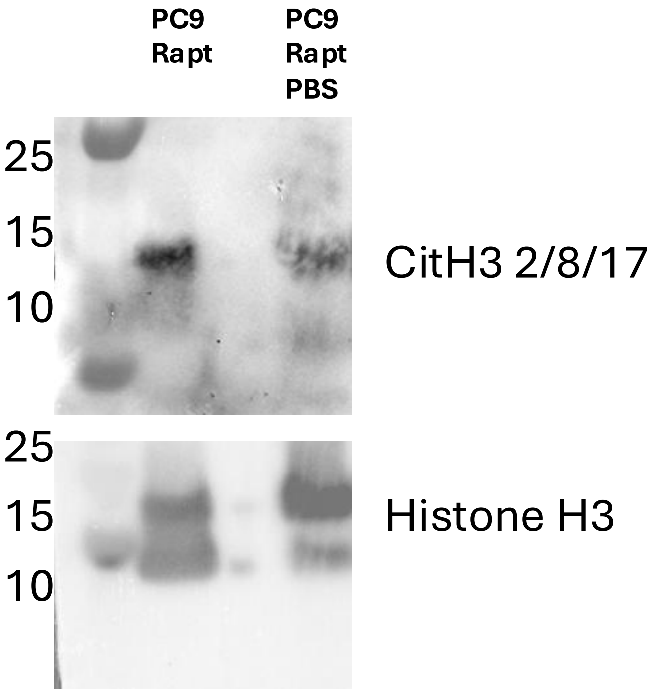

Supplement: Supplementary file 9 — Supplementary Material 9 [file 41598_2025_11807_MOESM9_ESM.pdf]
