# Supplementary figures and images for: Proteomic analysis of tumor cell nuclear expulsion reveals significant cell adhesion and RNA binding programs in extracellular chromatin
Source: Sci Rep. 2025 Aug 1;15:28054. doi: 10.1038/s41598-025-11807-z (PMC12317086; doi:10.1038/s41598-025-11807-z)

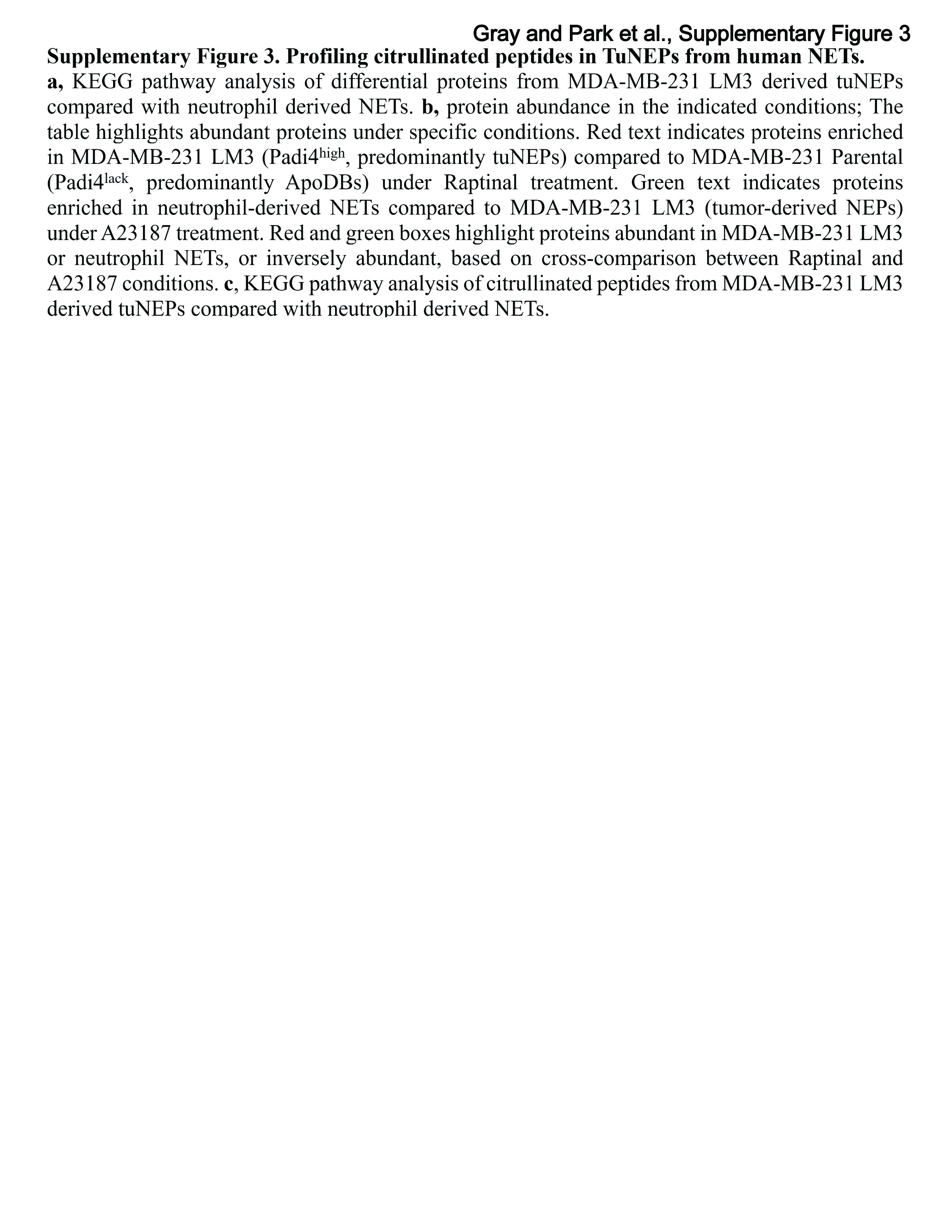

Supplement: Supplementary file 10 — Supplementary Material 10 [file 41598_2025_11807_MOESM10_ESM.tif]

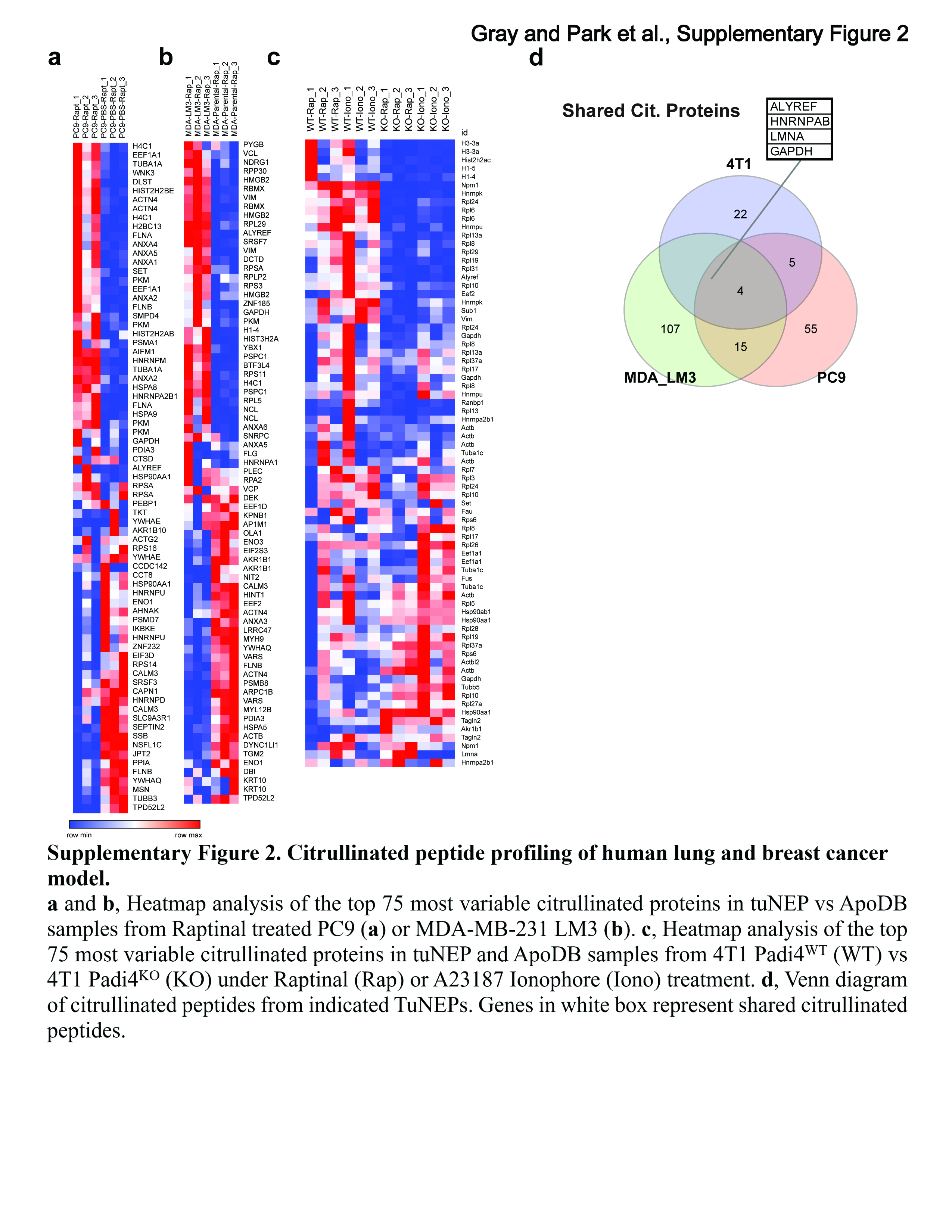

Supplement: Supplementary file 11 — Supplementary Material 11 [file 41598_2025_11807_MOESM11_ESM.tif]

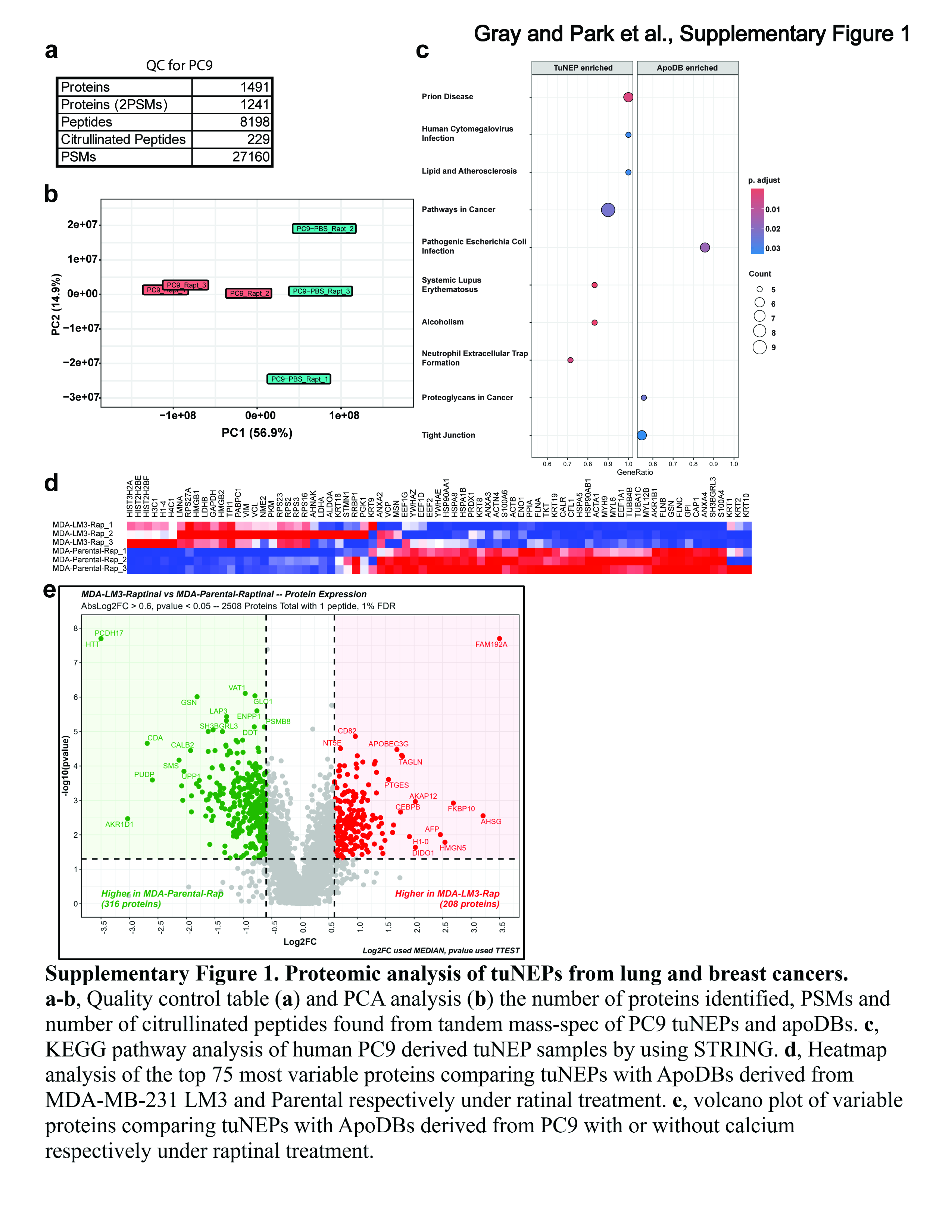

Supplement: Supplementary file 12 — Supplementary Material 12 [file 41598_2025_11807_MOESM12_ESM.tif]

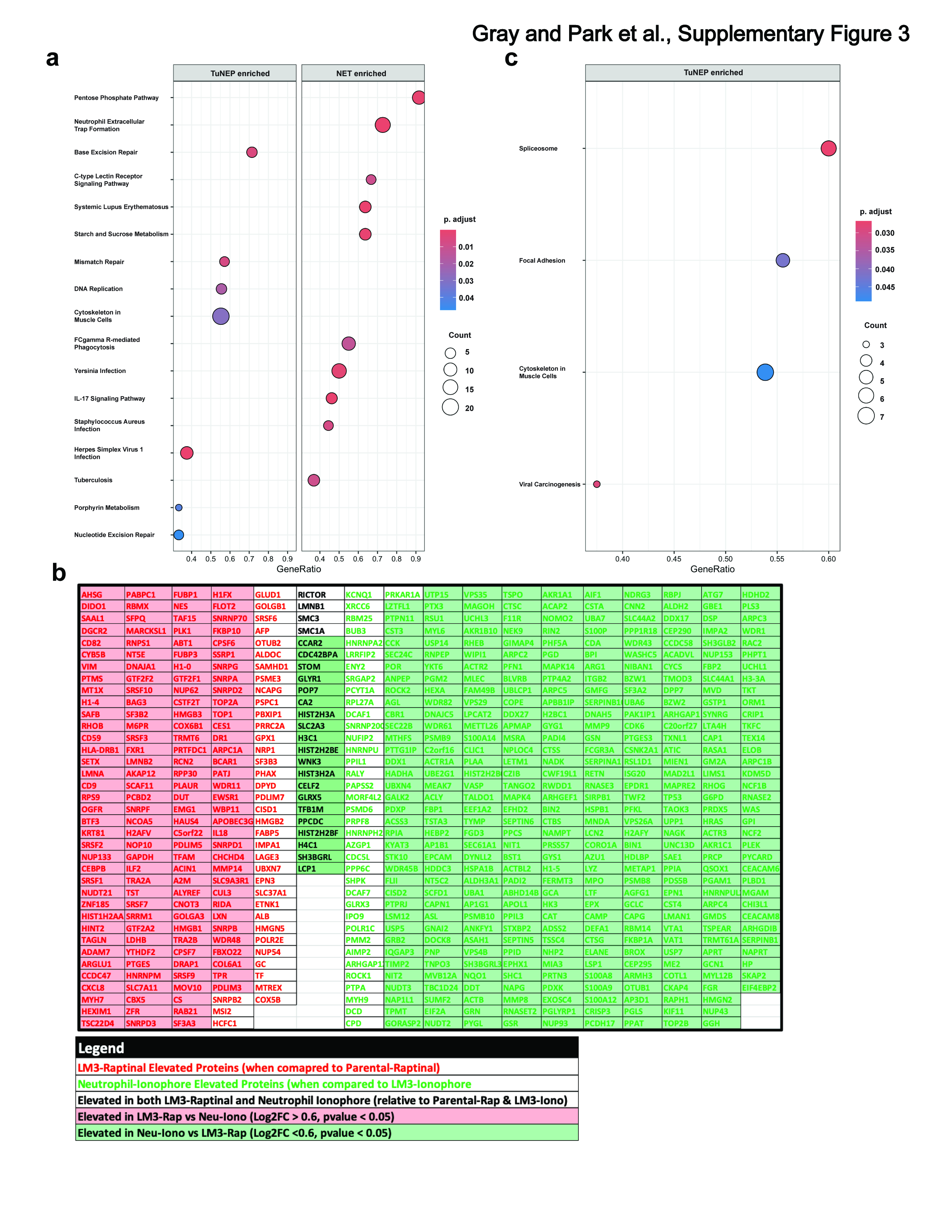

Supplement: Supplementary file 13 — Supplementary Material 13 [file 41598_2025_11807_MOESM13_ESM.tif]

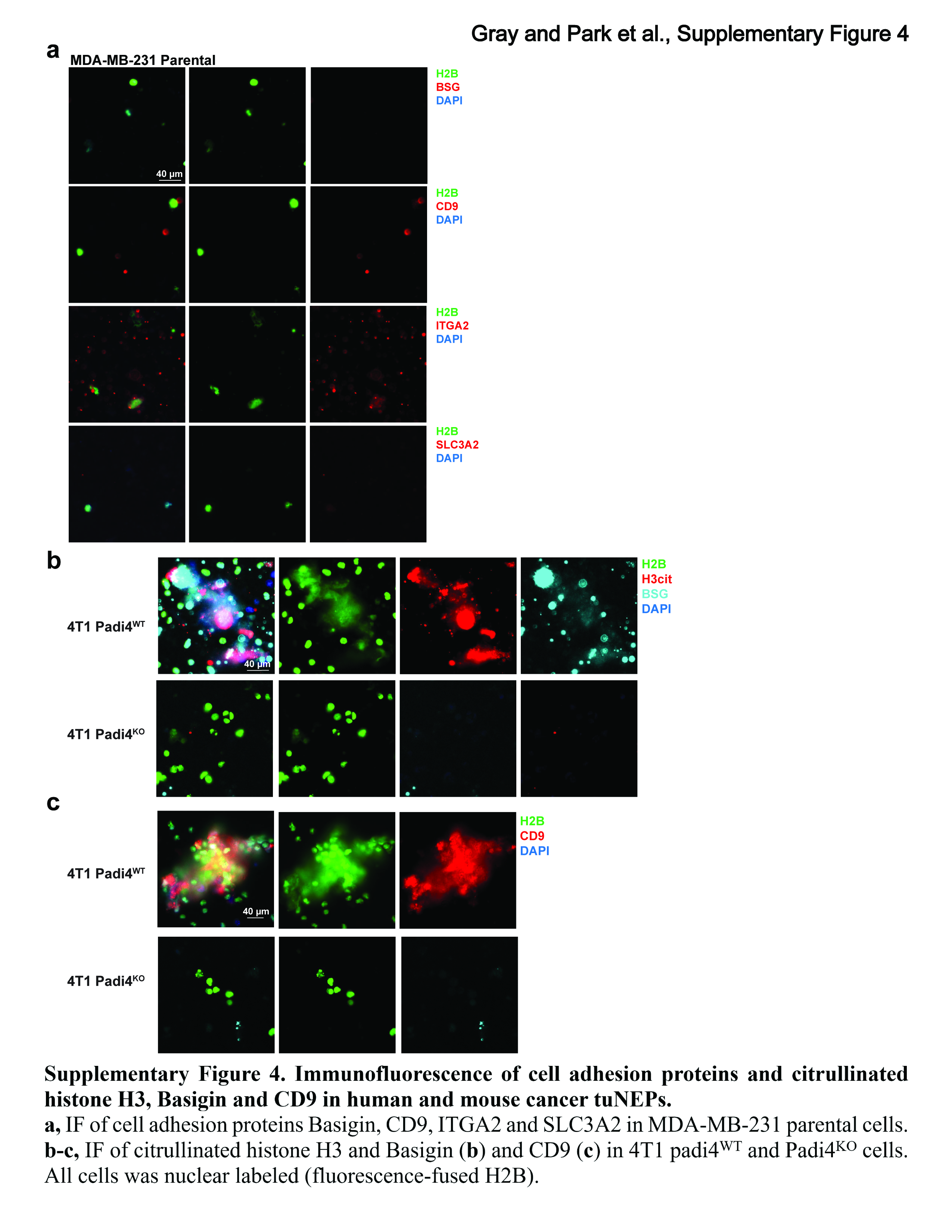

Supplement: Supplementary file 14 — Supplementary Material 14 [file 41598_2025_11807_MOESM14_ESM.tif]
